# An Overview of the Association between Schizotypy and Dopamine

**DOI:** 10.3389/fpsyt.2014.00184

**Published:** 2014-12-19

**Authors:** Christine Mohr, Ulrich Ettinger

**Affiliations:** ^1^Institute of Psychology, University of Lausanne, Lausanne, Switzerland; ^2^Department of Psychology, University of Bonn, Bonn, Germany

**Keywords:** schizotypy, personality, dopamine, cognition, psychopharmacology, neuroimaging, genetics

## Abstract

Schizotypy refers to a constellation of personality traits that are believed to mirror the subclinical expression of schizophrenia in the general population. Evidence from pharmacological studies indicates that dopamine (DA) is involved in the etiology of schizophrenia. Based on the assumption of a continuum between schizophrenia and schizotypy, researchers have begun investigating the association between DA and schizotypy using a wide range of methods. In this article, we review published studies on this association from the following areas of work: (1) experimental investigations of the interactive effects of dopaminergic challenges and schizotypy on cognition, motor control, and behavior (2), dopaminergically supported cognitive functions (3), studies of associations between schizotypy and polymorphisms in genes involved in dopaminergic neurotransmission, and (4) molecular imaging studies of the association between schizotypy and markers of the DA system. Together, data from these lines of evidence suggest that DA is important to the expression and experience of schizotypy and associated behavioral biases. An important observation is that the experimental designs, methods, and manipulations used in this research are highly heterogeneous. Future studies are required to replicate individual observations, to enlighten the link between DA and different schizotypy dimensions (positive, negative, cognitive disorganization), and to guide the search for solid DA-sensitive behavioral markers. Such studies are important in order to clarify inconsistencies between studies. More work is also needed to identify differences between dopaminergic alterations in schizotypy compared to the dysfunctions observed in schizophrenia.

## Introduction

Schizotypy, first coined by Rado (1), is a set of personality traits thought to mirror the subclinical expression of schizophrenia in the general population. Schizotypy encompasses behaviors, cognitions, and emotions resembling the signs and symptoms of schizophrenia. It is typically assessed in the general population using psychometric self-report questionnaires including, among others, the Schizotypal Personality Questionnaire (SPQ) (2), the Oxford–Liverpool Inventory of Feelings and Experiences (O-LIFE) (3), the Chapman scales (4), and the Rust Inventory of Schizotypal Cognitions (RISC) (5). Factor analytic studies have shown that schizotypy consists broadly of three subdimensions (3, 6). The *positive* schizotypy dimension describes perceptual aberrations resembling the hallucinations of schizophrenia as well as unusual views and ideas resembling the delusions of schizophrenia. The *negative* schizotypy dimension describes a loss of normal emotional, physical, and social functions such as the experience of pleasure or interest in social contacts. Finally, the *disorganized* dimension encompasses eccentric behavior and odd speech. Overall, the symptomatic phenomenology of schizotypy thus resembles the factor structure of symptoms reported in schizophrenia (7).

The literature tends to draw upon two scientific approaches toward the study of schizotypy, i.e., the quasi-dimensional and fully dimensional approaches. The quasi-dimensional, psychiatrically oriented approach, proposed by Meehl (8, 9), regards schizotypy as the subclinical expression of the symptoms of schizophrenia (10, 11). In that approach, schizotypal individuals are hypothesized to carry the genetic risk for schizophrenia. Evidence for this approach stems from some (12), but not all (13), taxometric studies. The fully dimensional approach (14), championed by Eysenck and Claridge (13, 15–18), regards schizotypy as a personality trait which is continuously distributed. It is assumed that schizotypal symptoms in the healthy population are qualitatively similar though quantitatively milder than those observed in schizophrenia. Evidence for the fully dimensional approach stems from taxometric studies that consider positive skewness of sample distribution (13). The high prevalence of psychotic-like experiences in the general population is also in accordance with the fully dimensional approach (19, 20). Importantly, both approaches emphasize the observation of variation in schizotypal features in the population. More work is, however, needed on the exact nature of the distribution and the boundary between non-clinical schizotypy and clinical schizophrenia (12, 13, 21, 22).

Irrespective of these open psychometric questions, biopsychological research on schizotypy is also based on the continuum assumption. There is considerable evidence that behavioral and brain correlates of schizophrenia are also related to schizotypy [for review, see, e.g., Ref. (11, 23, 24)]. By and large, the focus of these schizotypy studies has been on behavioral, cognitive, brain structural, and brain functional measures. Neuronal information transfer is, however, only possible with functional neurochemical transmissions at synapses. Therefore, neurochemical studies involving pharmacological challenges or molecular imaging methods are of utmost importance for our understanding of schizotypy. Most prominent so far are studies that link schizotypy to dopamine (DA). Comparable to above mentioned study domains, this link originates in the continuum assumption because schizophrenia has been linked to DA (25, 26), schizotypy might be associated with DA as well. In the following, we will explore this possible link. We will describe a selection of relevant psychopharmacological studies in patients with schizophrenia before referring to schizotypy studies that test the possible link to DA. First, however, we briefly introduce the structure and function of the DA system.

At this point, it should be mentioned that the schizophrenia spectrum also of course includes individuals with schizotypal personality disorder (SPD) and those at increased genetic or clinical risk for the illness. However, in the interest of focus, we restrict this overview to psychometrically identified schizotypy. Of course, it should be acknowledged that some, but not all, individuals with high levels of schizotypy also qualify for a diagnosis of SPD. For example, Raine (2) observed SPD in 55% of participants with SPQ scores in the top 10% of the distribution.

## The Structure and Function of Human Dopamine Systems

Neurotransmitters exert different actions throughout the brain; the type of action depends on the neurotransmitter, action site, and/or neuronal circuit (27). For instance, the amino acids GABA (inhibitory) and glutamate (excitatory) are found throughout the brain. Acetylcholine and monoamines (DA, norepinephrine, serotonin), on the other hand, are organized in systems. Systems imply that cell bodies producing these substances are located subcortically in anatomically circumscribed regions (27). In the case of DA, the nigrostriatal (important to motor control), mesolimbic (important to reward and motivation), and mesocortical (important to prefrontal functions) pathways have been distinguished on the basis of where their cell bodies are located and where their axons project. Moreover, two major DA receptor families have been identified, viz. the D1-like (D1 and D5) and D2-like (D2, D3, D4) receptors that are widely distributed throughout the brain. Because of the importance of the DA system to various mental health conditions, its complexity and functioning remain intensively investigated (28).

Of course, complex mental health disorders cannot be explained by single changes to complex neurotransmitter systems. Instead, subsystems are likely contributors to different psychopathologies. DA has indeed been associated with diverse neurological and psychiatric conditions such as Parkinson’s disease, schizophrenia, addiction, and attention deficit hyperactivity disorder [e.g., Ref. (29, 30)]. Also, neurotransmitter systems are not modular, i.e., they do not exert their actions in isolation. A given neurotransmitter system is in constant interaction with other systems. In schizophrenia, interactions between the DA and glutamate systems are intensively debated (31). Accordingly, pharmacological compounds can enhance DA release while also enhancing the release of the other two monoamines (norepinephrine, serotonin). The latter release might happen directly or indirectly through metabolism of DA into norepinephrine and the latter into serotonin (32, 33). This overall complexity in neurotransmitter systems highlights the fact that individual psychopharmacological studies have inherent limitations in investigating the link between schizotypy and DA. As exemplified in the following sections, we depend on findings from a multitude of studies using different but complementary approaches.

## Dopaminergic Effects in Patients with Schizophrenia

Neuroleptics were first discovered in the early 1950s (34). Since their discovery, it has been shown that these DA antagonists ameliorate acute psychotic symptoms [e.g., Ref. (35, 36)]. It is still accepted that DA is key in the treatment and pathophysiology of schizophrenia (37–39). For example, patients with schizophrenia profit from DA antagonistic treatment (38) and show symptom worsening after DA agonistic treatment (40, 41). In healthy populations, DA agonists can trigger psychotic or psychosis-like symptoms (42, 43). Such findings led to the suggestion that a hyperactive DA system results in acute psychotic symptoms (25, 38). Davis et al. (25) further elaborated on this suggestion by proposing DA abnormalities to depend on the brain region, i.e., D1 receptors being predominantly found cortically and D2 receptors subcortically. They specified that frontal hypodopaminergia would result in striatal hyperdopaminergia. Regarding symptoms, they related negative symptoms to frontal hypodopaminergia and positive symptoms to striatal hyperdopaminergia.

Howes and Kapur (44) refined this previous DA theory by suggesting that multiple “hits” (e.g., social, environmental, and cultural stressors) result in DA dysregulation at the pre-synaptic level. Such DA dysregulation is hypothesized to lead to changes in how events are appraised, potentially because events become overly salient (i.e., attract increased allocation of meaning). According to these authors, an alteration in pre-synaptic DA is the final common pathway to psychosis and psychosis-proneness (45). Yet, other evidence points to the role of post-synaptic processes (46). Very recently, Seeman (39) outlined how dynamic changes of post-synaptic D2 receptors might contribute to symptoms experienced in schizophrenia. Mainly based on animal studies, Seeman reports on D2 receptors that can either take a high affinity state for DA (D2High) or a low affinity state for DA (D2Low). Reviewing the literature, he suggests that psychosis is associated with the more active and normally less common D2High state.

Overall, despite various suggestions on the involvement of alternative neurotransmitter systems in psychosis and, by inference, schizophrenia (31, 47), recent notions highlight that DA is the likely final common pathway to psychosis. The precise mechanisms and sites of action remain inconclusive, and continued research reveals ever more complex synaptic dynamics such as the ones described for the D2High and D2Low affinity states. It remains to be clarified whether such alterations of the DA system are specific to overt clinical psychosis or are also evident, though less pronounced, along the schizophrenia spectrum including healthy schizotypy. Howes and Kapur (44) previously reviewed a number of studies showing an involvement of DA in the prodromal state, in ultra-high risk populations, in relatives of schizophrenia patients and in schizotypy. Because these populations lie on the psychosis continuum, part of these and additional studies will be discussed in more detail.

In an 18F-dopa positron emission tomography (PET) study, Howes et al. (48) observed that patients with prodromal psychotic symptoms showed enhanced striatal 18F-dopa uptake, intermediate to that of patients with schizophrenia and healthy controls. This enhanced uptake correlated positively with prodromal symptomatology and neuropsychological impairments [but see in Ref. (49)]. In a 3-year follow-up study, Howes et al. (50) observed that greater striatal DA synthesis capacity was observed in a psychosis transition group as compared to a group that did not transit into psychosis. Moreover, this capacity in the transition group correlated positively with symptom severity. In another study, elevated pre-synaptic striatal uptake was comparable between patients with SPD and remitted patients with schizophrenia (51). It was also elevated for individuals at ultra-high risk for psychosis, i.e., those who show impaired socio-occupational functions and attenuated psychotic symptoms (e.g., perceptual abnormalities, paranoia) (52). An increase of striatal pre-synaptic DA synthesis has also been observed in first-degree relatives of patients with schizophrenia (53). Relatives additionally showed an altered stress response, demonstrated by increased levels of plasma DA metabolites when compared to healthy controls (54). This increase was associated with psychotic-like symptoms subsequent to daily stressors (experience sampling methodology) (55). In yet another study, psychotic patients and unaffected relatives released significantly more DA in striatal regions subsequent to the inhalation of delta-9-tetrahydrocannabinol (the main psychoactive ingredient of cannabis) than healthy controls (56). In sum, these studies show that DA abnormalities are not restricted to individuals with a clinical diagnosis of schizophrenia, but are also evident in the extended phenotype. Findings specific to schizotypy are presented further below.

## Methods in the Study of Dopamine and Its Relation to Schizotypy and Cognition

Various methods are available to measure aspects of the DA system in healthy humans. The most promising to study the link between schizotypy and DA include (1) experimental pharmacological challenges of the DA system, (2) neurobiologically informed, DA-sensitive cognitive and behavioral measures, (3) molecular studies of genes involved in DA neurotransmission, and (4) molecular neuroimaging of the DA system. Here, we briefly outline these methods. In the next section, we discuss how they have been used in work on schizotypy.

The first methodological approach directly tests the link between schizotypy and DA. For instance, one could test DA agonistic and antagonistic treatment effects on the experience of schizotypy as well as related correlates in healthy populations. This approach corresponds to tests of medication effects in patients. In patients, however, pharmacological treatment consists of the daily administration of medication over many weeks, given that the clinical antipsychotic effects of such compounds take several weeks to emerge (57). When testing healthy populations, such long-term drug administration would be unethical and potentially dangerous. For instance, antipsychotic treatment causes severe side effects [e.g., Ref. (58) for a meta-analysis]. These and other (e.g., economic and clinical) factors explain why the investigation of long-term medication effects is common in clinical studies while single (or limited) short-term drug effects are frequently investigated in healthy volunteers.

Importantly, short-term dopaminergic drug applications directly modulate the DA system, thereby allowing the evaluation of dopaminergic effects on domains of cognition, emotion, motor control and their neural correlates. Such studies tend to take place in well-controlled laboratory settings using tight methodological controls, such as double-blind procedures and placebo conditions [e.g., Ref. (59–61)]. Such paradigms inform on acute drug effects and their interaction with schizotypy. They also allow addressing clinical questions hard to implement in patients (40–43). As outlined above, however, it should be borne in mind that the DA system does not work in isolation but instead interacts with other neurotransmitter systems; therefore, the specificity of any pharmacological effect on behavior and cognition remains to be characterized further.

The second methodological approach involves the assessment of DA-mediated cognitive and behavioral performance measures and their covariance with schizotypal personality traits. This approach is indirect in comparison to the first approach. Yet, the use of DA-mediated performance measures is popular. Various paradigms have been drawn upon (62–67). Of importance, previous studies have shown that these performance measures are sensitive to modulations of the DA system. In particular, such measures are affected by dopaminergic drugs in humans [e.g., Ref. (61, 68, 69)], human mental health conditions in which the DA system is affected [e.g., Ref. (64, 65)] and by experimental dopaminergic interventions in animals [e.g., Ref. (64, 69)].

The third methodological approach involves molecular imaging methods. As indicated above, methods such as PET and single photon emission computed tomography (SPECT) can provide *in vivo* data on markers of the DA system (such as DA synthesis or receptor and transporter availability) in the human brain (45). These markers can be quantified and related directly to schizotypy scores. As will be detailed below, PET and SPECT methods have recently been applied to study the DA system as a function of schizotypy.

The fourth methodological approach involves the exploration of the role of DA in schizotypy by studying statistical associations of schizotypy scores with variants in genes whose protein products are known to be involved in the DA system (70, 71). Such genes may include those coding for receptors, transporters or enzymes involved in DA synthesis, release, reuptake, degradation or any other aspect of DA transmission. The effects on DA transmission of such genetic variants, when known, allow drawing conclusions about differences in DA transmission in relation to individual differences in schizotypy.

## Dopamine and Schizotypy: Empirical Evidence

A role of DA in schizophrenia and schizotypy has long been postulated (67, 72–74). Empirical evidence on the nature of the association between DA and schizotypy has been scarce and the association has rarely been investigated directly (68, 75). However, studies have accumulated over the last 15 years. Here, we give an overview on the outcome of these studies, irrespective of which methodological approach has been applied. In case that DA was investigated indirectly (the second methodological approach mentioned above), we distinguish between studies that assessed more basic behavioral functions and those that assessed higher cognitive functions. Often these studies added a direct manipulation of the DA system using the first methodological approach outlined above. Therefore, we discuss these together with the purely behavioral data on simple or complex behavioral functions. In the case of molecular genetic and molecular imaging studies, many findings are based on associations with questionnaire scores. We discuss these studies separately.

### Basic behavioral functions

Turning behavior is a behavioral marker of a relatively hyperactive DA system in one over the other hemisphere. Animal studies [Ref. (76) for overview] and a study testing patients with asymmetrical Parkinson’s disease (77) showed that whole-body turning occurs away from the hemisphere with the more active DA system. Acutely psychotic patients (65) and individuals with elevated positive schizotypal features (74) yielded a preference for left- over right-sided turns pointing to a relative hyperactive right-hemispheric DA system along the schizophrenia spectrum. In a study that directly tested the influence of DA on turning behavior, half of the participants received a placebo and the other half a non-specific DA agonist (levodopa) (78). Turning biases were investigated as a function of participants’ positive and negative schizotypal features. The authors found that elevated positive schizotypy associated with a left- over right-sided turning preference in the placebo group. In this same group, negative schizotypy associated with a right- over left-sided turning preference. In the levodopa group, these relationships were not strengthened, but actually reversed. The authors argued that a higher than normal DA availability might have balanced out pre-existing DA asymmetries. In another experiment on the same participants, schizotypy and levodopa treatments were unrelated to lateral turning biases in a computer-based object-rotation task (66).

Spontaneous eye blink rate (SBR) is indicative of cerebral DA activity. Eye blinks occur spontaneously, frequently, and mostly without awareness. They are primarily important for the health of the eye surface and clarity of vision. Decades ago, SBR has been suggested to be a sensitive marker of striatal DA activity (64). SBR has been associated with changes in concurrent cognitive processes, perceptual load, and level of arousal (79, 80). It is enhanced in schizophrenia, in particular in the unmedicated or drug-naïve state (64, 81, 82), and decreases with antipsychotic treatment (64, 82, 83). In line with Davis et al.’s (25) notion of a link between negative symptoms and hypodopaminergia on the one hand and positive symptoms and hyperdopaminergia on the other hand, negative symptoms were associated with a decreased SBR (83) and positive symptoms with an increased SBR (82, 83). Additionally, SBR increased with a DA agonistic treatment in healthy individuals (84, 85).

In a dopaminergic challenge study, half of the participants received a placebo while the others received an unspecific DA agonist (levodopa) (86). SBR did not differ between substance groups, but correlated positively with negative (but not positive) schizotypy scores after levodopa intake. The authors conjectured that this effect occurred because negative schizotypy is *a priori* related to hypodopaminergia. In another study, however, SBR correlated positively with participants’ psychoticism scores, a subdimension of the Eysenck Personality Questionnaire (87). Some authors argued that psychoticism forms part of the schizotypy spectrum (88). Other authors linked psychoticism to impulsiveness, lack of cooperation, rigidity, low superego control, low social sensitivity, low persistence, lack of anxiety, and lack of feelings of inferiority (89) – features associated with borderline and antisocial personality disorder rather than with the schizophrenia spectrum (90, 91). The psychoticism dimension seems indeed the least clear-cut dimension of the three Eysenckian dimensions (90, 91). Whatever the psychoticism dimension is measuring, the latter SBR finding suggests that psychoticism is probably more sensitive to the DA system subserving SBR than is schizotypy (or negative schizotypy) as such.

Stereotyped behavior is thought to be sensitive to DA. It is observable in schizophrenia (92, 93) and after amphetamine consumption in healthy individuals (69). In schizophrenia, stereotyped behavior occurs on the motor (e.g., walking backwards and forwards, repetitive jaw movements) (94) and higher cognitive level. For instance, perseverative errors in the Wisconsin Card Sorting Test are elevated in schizophrenia [Ref. (95) for overview], SPD (96, 97) and as a function of both high positive and negative schizotypy (98, 99). Random number generation tasks show that patients with schizophrenia (100, 101), healthy participants after amphetamine administration (69), and healthy positive schizotypal participants (102, 103) yield stereotypical response biases when compared to respective controls. Finally, when rotating figures into advantageous target positions on a computer screen, left or right turns had to be applied dynamically to obtain maximal scores (66). Sticking stereotypically to one or the other direction would be disadvantageous. The latter study reported on two experiments with one being a between-subject levodopa placebo-controlled experiment. The authors observed that individuals with relatively high as compared to low positive schizotypy were behaving more stereotypically. Yet, in the levodopa group, high positive schizotypal individuals performed less stereotypically than the low positive schizotypal individuals. Comparable to the findings on turning behavior, results seem to suggest that a higher than normal DA availability in positive schizotypes balances out pre-existing DA-mediated behavioral abnormalities.

Prepulse inhibition (PPI) is another DA-sensitive measure that has been studied in relation to schizotypy. PPI is a cross-species phenomenon that refers to a reduction in the amplitude of the startle response to a strong sensory stimulus, the pulse, if this stimulus is preceded by 30–500 ms by a weak stimulus, the prepulse (104). The weak prestimulus is thought to evoke inhibitory mechanisms, which limit further stimulation until the processing of the prepulse has been completed, resulting in a reduced impact of the pulse. PPI is thought to reflect an early sensory gating process to avoid interference from simultaneous processing of several stimuli. It is, thus, a largely automatic measure of inhibitory function. Experimental studies in animals have shown that a cortico-striato-pallido-thalamic circuitry underlies PPI (105).

Patients with schizophrenia (106) and SPD (107) show reproducible reductions in the magnitude of PPI, which is thought to lead to sensory overstimulation causing cognitive and behavioral confusion. Also, PPI deficits are induced in numerous experimental models of schizophrenia through, e.g., isolation rearing (108), ketamine (109), sleep deprivation (110, 111), and phencyclidine (112). The robust and reliable PPI reduction in schizophrenia can be restored, at least partially, with antipsychotic treatment, with some advantage of atypical compounds over first-generation, typical antipsychotics (113–119).

Importantly, reduced PPI has been observed in relation to elevated SPQ total, cognitive-perceptual and interpersonal scores (120), Minnesota Multiphasic Personality Inventory (MMPI) psychosis-proneness scores (121) and Eysenckian psychoticism scores (122). Evidence from a functional magnetic resonance imaging (fMRI) study of PPI points to reduced activation in the insula, putamen, thalamus, inferior parietal cortex, hippocampal gyrus, and fusiform gyrus in relation to higher levels of Eysenckian psychoticism scores (122). These BOLD patterns show some resemblance with the data obtained from samples of patients with schizophrenia (123). Of relevance to the present discussion, areas such as the putamen are rich in DA receptors, consistent with the hypothesis that PPI is at least in part dopaminergically mediated (124–126).

A final basic DA-mediated paradigm is latent inhibition (LI) (62, 127, 128). Comparable to PPI, LI is a cross-species phenomenon, which is sensitive to pro- and antidopaminergic challenges (62). LI refers to the finding that non-reinforced pre-exposure to a stimulus, that is later to be conditioned, causes less efficient conditioning when the same stimulus is subsequently reinforced. This phenomenon has been observed in both humans and animals. LI has also been investigated in relation to schizophrenia and schizotypy. A recent review showed reduced LI in the acute phase of patients with schizophrenia and preserved LI in chronic schizophrenia (129). That review also found a modest but relatively consistent relationship between schizotypy and LI, particularly of positive schizotypy (130). In particular, higher levels of positive schizotypy seem to be associated with less LI in healthy participants. These studies, together, suggest an involvement of dopaminergic alterations in the LI deficits in schizophrenia and positive schizotypy.

### Higher cognitive functions

Dopamine is thought to modulate the signal-to-noise ratio in semantic networks (131, 132). The reduction of prefrontal tonic dopaminergic modulation (hypofrontality) in schizophrenia is assumed to decrease the contrast between a signal and noise. This decreased contrast leads to a disinhibited spreading activation within semantic networks [see in Ref. (132) for an overview]. This increased spreading activation would result in remote associations (psychotic symptom) and enhanced semantic priming effects (laboratory measures) in schizophrenia (132). In healthy populations, on the other hand, an experimentally enhanced DA availability (levodopa consumption) focused such priming effects (133). Yet, this dopaminergic modulation of semantic networks differed for the two cerebral hemispheres with a hyperdopaminergia being more prominent in the right than left hemisphere (see previous section). This hyperdopaminergia (potentially resulting from a frontal hypodopaminergia) has been considered to explain the observation that enhanced spreading activation was more relevant for the right than left hemisphere in schizophrenia (134) and healthy individuals high in positive schizotypy (60, 135). Independent studies suggested that these DA-mediated functions explain both these lateralized priming effects and enhanced remote associative processing more generally (136, 137) as well as right-hemisphere shifts of functions in a broader sense. Indeed, the left hemisphere seems compromised in patients with schizophrenia with DA antagonists restoring if not reversing such altered hemispheric asymmetries (138–143).

Studies showed that lateralized lexical decision performance is relatively biased toward the right hemisphere in high as compared to low scorers on a positive but not negative schizotypy questionnaire (144, 145). In a pharmacological challenge study, superior left hemisphere language dominance was associated with elevated negative schizotypy scores in a levodopa (but not placebo) group (145). Thus, levodopa might restore left hemisphere language dominance in healthy individuals through (i) the attenuation of the contribution of the right hemisphere to language as a function of positive schizotypy and (ii) an increased contribution of the left hemisphere as a function of negative schizotypy. In an independent sample, but using the same task, the authors calculated signal detection theory measures of sensitivity (d-prime) and response tendency (criterion) (146). Results showed lower d-prime in the levodopa than in the placebo group in individuals low in positive schizotypy. For the response criterion, individuals in the placebo group showed a loosened versus conservative response criterion when being high versus low in positive schizotypy. In the levodopa group, these response tendencies were attenuated. This study suggests that a higher than normal DA availability (i) reduces d-prime in low positive schizotypy individuals and (ii) turns low positive schizotypy individuals less conservative and high positive schizotypy individuals more conservative in their response behavior.

Higher cognitive functions such as working memory and cognitive control have also been studied using experimental psychopharmacological designs. Using a between-subjects, double-blind, placebo-controlled design with groups of high and medium total SPQ scores, amisulpride, a clinically effective antipsychotic with strong D2/D3 receptor antagonistic action, improved working memory and verbal fluency performance in the schizotypy group but impaired it in the medium schizotypy control group (59). The same multi-center study investigated the effects of risperidone, a clinically effective atypical antipsychotic with action on D2 as well as 5-HT receptors, on performance on the antisaccade task, a prominent measure of response inhibition (147, 148). It was found that risperidone impaired antisaccade performance in the medium schizotypy group, whereas performance in the high schizotypy group showed a non-significant tendency toward improvement (149). These findings are in agreement with previous evidence of risperidone impairing antisaccade performance in healthy controls (150) but improving it in patients with schizophrenia (151, 152).

To summarize, findings of this section suggest that individuals with high levels of schizotypy may benefit from DA agonists in terms of cognitive performance. In contrast, individuals with low levels of schizotypy may deteriorate in cognition with DA agonists. Concerning DA antagonists, people with high levels of (particularly positive) schizotypy may benefit in cognitive function from DA antagonistic compounds similar to patients with schizophrenia, or at least tolerate them. People with low or medium levels in (particularly positive) schizotypy may become impaired in these functions. Overall, given the role of DA in the effects of clinically effective antipsychotics (153), the presented data indicate that DA impacts some of the cognitive deficits observed in high (mainly positive) schizotypy (24, 78, 154). However, antipsychotics such as risperidone do not act only on the DA system, thereby limiting the implications for a dopaminergic basis of schizotypy (149).

### Schizotypy and dopamine system genes

Twin studies have established that individual differences in the various subdimensions of schizotypy in the general population are to a significant part explained by additive genetic factors. Heritability estimates are in the range of 30–50% [see, e.g., Ref. (155–157)], although not all studies have found significant heritabilities (158). These behavioral genetic studies, thus, provide a basis for molecular genetic investigations such as those specifically targeting candidate genes related to the DA system.

The most frequently studied gene in the context of schizotypy is the gene coding for catechol-*O*-methyltransferase, the *COMT* gene (159–161). These studies were originally informed by significant associations of a single nucleotide polymorphism (SNP) in the *COMT* gene with schizophrenia (162); however, later meta-analyses have suggested that this association is not significant (163, 164). The COMT enzyme degrades catecholamines including DA in the synaptic cleft. Due to the paucity of the DA transporter in the prefrontal cortex, COMT plays a particularly prominent role in degrading pre-synaptic DA in that part of the brain. The *COMT* gene (located in chromosome 22q11.1-q11.2) contains a G to A missense mutation, resulting in a substitution of methionine (met) for valine (val) at codon 158 of the membrane-bound isoform of the protein (reference sequence identification code rs4680). This allelic variation (val158met) is a functional polymorphism: The met158 allele has about one third to one fourth of the activity of the val158 allele, resulting in less efficient catecholamine catabolism and, therefore, higher DA levels.

A number of published studies have reported associations between schizotypy and the *COMT* val158met polymorphism in healthy subjects. A series of publications from a large-scale study of apparently healthy young men, the ASPIS study, has provided evidence that the high-activity Val allele may be associated with schizotypy. In particular, the Val allele was found to be associated with elevated total SPQ and Perceptual Aberration Scale scores in a first analysis of 379 subjects (165). In a subsequent study using an extended sample of 543 individuals and a factor analysis of SPQ into four factors (cognitive-perceptual, negative, disorganization and paranoia) the Val loading was associated with increased total SPQ and increased negative and disorganized factor scores (71). In a further extension of 908 subjects where the analysis was also performed for individual SPQ dimensions, an association of Val with increased total SPQ, negative disorganized, and paranoia factors was observed. Additionally, there were specific relations of Val loading with increased scores in magical thinking, constricted affect, and odd speech subscales of the SPQ (166).

Broadly confirming this evidence, Grant et al. (167) observed in an independent sample of 280 healthy volunteers that Val/Val homozygotes had significantly higher positive schizotypy scores (O-LIFE unusual experiences) than Met carriers.

Other studies obtained evidence of an association between the Val allele and increased schizotypy in samples of relatives of psychiatric patients. One study (168) observed that among 81 first-degree relatives of schizophrenia patients the Val allele was associated with high negative schizotypy (social and physical anhedonia), while no association was found in 38 relatives of patients with bipolar disorder or in 30 healthy controls. Schürhoff et al. (70) studied *COMT* rs4680 and schizotypy in a combined sample of relatives of schizophrenic patients, relatives of bipolar patients and healthy controls (total *N* = 106). The authors observed higher total, positive and negative SPQ schizotypy (but not disorganization) scores in association with an increasing number of Val alleles.

Overall, these studies suggest that the Val allele is associated with higher levels of various schizotypy subdimensions. However, evidence to the contrary as well as failures to replicate have also been published. For example, Sheldrick and colleagues (169) reported higher SPQ-B Disorganization scores in the *COMT* Met/Met group than in the Val/Val group (*N* = 522 volunteers). Also, no significant association of overall schizotypy with rs4680 genotype was observed in individuals with velo-cardio-facial syndrome (170) or in a mixed sample of members from families with bipolar disorder as well as unaffected controls (total *N* = 222) (171). Ma and colleagues (172) similarly reported a lack of significant associations between rs4680 and SPQ total and subscale scores in 465 Chinese participants. Finally, Ettinger et al. (173) found no statistically significant association of positive schizotypy (RISC) with rs4680 in a small sample of healthy males (*N* = 31).

These inconsistencies remain to be resolved, both through additional original studies and a comprehensive meta-analysis that also addresses potential moderator variables. A number of factors may play a role, including the choice of schizotypy questionnaire (174) as well as the ethnic and sociodemographic composition of the sample (175). Other moderating variables may also be important. For example, the above mentioned study by Savitz and colleagues (171) observed, in the absence of an overall association of rs4680 with STA schizotypy, that there was an interaction between childhood trauma (as measured with the Childhood Trauma Questionnaire) and rs4680 on STA: The genotype was associated with STA only in individuals with higher trauma scores, such that Val/Val individuals showed increasing STA with increasing trauma, whereas other genotype groups did not. The possibility of interactive effects of genes and the environment is intriguing and needs to be examined in more detail.

In addition to *COMT*, a number of other DA-related genes have been investigated in relation to schizotypy. Ettinger et al. (173) obtained no evidence of significant associations of positive schizotypy (RISC) with polymorphisms in the *DRD3, DRD4*, and *SLC6A3* genes in a sample of 31 healthy subjects. Grant et al. (167), however, reported significant associations of different O-LIFE subscales with *DRD2 Taq1A*, *MAOA*-uVNTR and *SLC6A3* in 288 participants. Finally, a recent study by Taurisano et al. (176) observed an association between SPQ total score and *DRD2* rs1076560 in 67 subjects, with GT heterozygotes showing higher scores than G allele homozygotes.

Overall, the discussed molecular genetic findings are promising but must be considered preliminary; they require replication and further investigation in larger samples. Genetic research has the possibility of informing the molecular basis of inter-individual variation in schizotypy and as such makes a unique, non-invasive contribution to the neuroscientific method arsenal available to schizotypy researchers. An additional appeal of this method is the possibility of combining genetic data with other neuroscience methods, such as functional and structural neuroimaging (177).

### Molecular imaging of the dopamine system in relation to schizotypy

Measures of the DA system in humans can also be obtained *in vivo* using molecular imaging techniques such as PET and SPECT. These methods have been applied successfully to the study of the dopaminergic basis of schizophrenia (45). In contrast, relatively few PET or SPECT studies have investigated the relationship between DA system markers and schizotypy.

An early ^123^I-IBZM single photon emission tomography investigation (178) reported a significant *negative* relationship between Eysenckian psychoticism scores and D2 receptor binding in the basal ganglia (relative to frontal cortex) in a small sample of healthy volunteers. Extending this first evidence, a [^11^C]raclopride PET study (179) found lower D2 receptor density in putamen to be associated with higher scores on the Karolinska Scales of Personality detachment scale, a measure tapping aspects of negative schizotypy. Breier and colleagues replicated this finding, reporting a negative association between the Karolinska detachment scale and striatal D2 receptor binding using [^11^C]raclopride PET in an independent sample of healthy volunteers (180).

In contrast, a more recent SPECT study observed a positive relationship between the disorganization score of the SPQ and D2/3 receptor binding in the right striatum (relative to occipital cortex) (181). Most recently, Taurisano et al. (176) used SPECT and found a positive correlation between D2 receptor binding in the right putamen and total SPQ score in individuals heterozygous for the *DRD2* rs1076560 genotype but not in individuals homozygous for the G allele, suggesting interactive effects of genotype and D2 availability on schizotypal personality. No association between whole striatum DA synthesis capacity and total SPQ score was observed in a PET study of subjects with persistent auditory verbal hallucinations (182).

A different line of molecular imaging studies has focused on dopaminergic challenges in relation to schizotypy. It has previously been shown that patients with schizophrenia, both in the acute phase and in remission, exhibit amphetamine-induced reductions in D2 and D3 DA receptor binding potential in the striatum (40, 183). This result suggests increased striatal DA release in schizophrenia and complements the evidence of heightened pre-synaptic DA function in this disorder (45). Interestingly, a similar pattern of increased striatal amphetamine-induced DA release has been observed in SPD (51). Further evidence confirming and extending this pattern across the schizophrenia spectrum comes from a [^18^F]fallypride PET study (184). That study showed significant correlations between striatal DA release after a single administration of amphetamine and SPQ total and disorganized dimension scores in healthy volunteers (184). Together, these studies provide evidence for a common striatal dopaminergic dysregulation in both schizotypy and schizophrenia.

Finally, based on evidence of stress as a possible risk factor for schizophrenia (185), molecular imaging methods have been employed to investigate the effects of experimentally induced stress on DA turnover as a function of schizotypy. Using [^11^C]raclopride PET, it was shown that participants with high levels of negative schizotypy – but not controls or participants with high levels of positive schizotypy – showed a significant stress-induced striatal reduction in binding potential (186).

Overall, studies using molecular imaging methods such as PET or SPECT are able to provide relatively direct markers of the DA system *in vivo* and, as such, can be used profitably to investigate its relationship not only to schizophrenia but also to schizotypy. However, evidence of associations between DA markers and levels of schizotypy in healthy participants is still scarce. To summarize, while there are associations between lower D2 receptor availability and higher psychoticism and detachment, there is also a report of an association between lower D2/3 receptor binding and lower disorganized dimension schizotypy. This pattern may imply differential associations of DA levels with different dimensions of schizotypy. Finally, there is evidence of increased striatal DA release following amphetamine administration or stress induction in schizotypy.

## Conclusions

In this overview, we discussed studies linking DA and schizotypy. We first introduced the neurotransmitter DA and sketched some *in vivo* methods of its study in patients with schizophrenia and healthy controls. We presented evidence from the behavioral literature showing that high levels of schizotypy (in particular positive schizotypy) are associated with DA-sensitive functions, in line with behavioral tendencies previously reported in schizophrenia. In addition, we presented evidence that both increased (DA agonists) and decreased (DA antagonists) DA availability seems to be beneficial to behavioral deficits in high schizotypes (mainly positive schizotypy). Those low in schizotypy, however, seemed to deteriorate in their behavioral performance following antipsychotic treatment. While only a careful conjecture, it seems that an increase as well as a decrease of DA may restore function in high schizotypes but deteriorate it in low schizotypes. Overall, we conclude that variance in schizotypy may be explained in part by alterations to the DA system. Of course, as noted, the DA system does not act in isolation but in constant and complex interactions with other neurotransmitter systems.

An underdeveloped theoretical point not only here, but also in schizotypy research more generally, is a well-recognized framework integrating empirical evidence on links between DA and schizotypy from various levels of analysis (not only concerning DA) into a coherent theory. Important theoretical contributions by Meehl (8, 9) and others (16, 187) have influenced our current thinking on schizotypy. A significant body of new data has been amassed that needs to be theoretically linked. In particular, we think of the psychopharmacological, imaging and genetic findings that are based on techniques that had not been available before. To achieve this aim, it may be instructive to turn in two directions for inspiration, viz. in the direction of (i) personality theory in individual differences research and (ii) clinical schizophrenia research.

Regarding individual differences, DeYoung (188) recently integrated previous assumptions into a theoretical framework that is relevant to our discussion of the role of DA in schizotypy. Particularly relevant is his discussion of “apophenia,” i.e., individuals’ tendency for overinclusive thinking and perception. Conrad (189) termed apophenia as the “unmotivated seeing of connections” accompanied by a “specific experience of an abnormal meaningfulness” (p. 46). For instance, one can refer to apophenia when individuals see and create pattern in random noise, whether this noise is perceptual (190, 191), semantic (136), or probabilistic (192). The suggestion of a link between apophenia and positive schizotypy in particular (136, 193) including its modulation by DA (60, 194) is not new. However, DeYoung (188) links apophenia not only with positive schizotypy and the positive symptoms of psychosis but adds another link, namely that apophenia can be predicted by openness, one of the personality traits of the five factor model of personality (195). Accordingly, apophenia can be called “openness to implausible patterns” [Ref. (188); p. 13]. This openness and its link to apophenia are hypothesized to stem from high-activity levels in the dopaminergic salience system. As mentioned above (44), recent theories on the role of DA in schizophrenia suggest that stressors dysregulate DA at the pre-synaptic control level. This is hypothesized to lead to changes in the appraisal of events, making them more salient (i.e., causing apophenia). Thus, DeYoung’s (188) DA theory of personality is of interest as it ties schizotypy in with other personality traits and establishes parallels with schizophrenia at the cognitive (overinclusive pattern detection) and neurophysiological (DA) level.

These parallels link directly to the second direction of inspiration, i.e., clinical schizophrenia research. Here, aberrant salience has become a predominant topic. Current theorizing has provided promising explanations as to how DA links with the symptomatic expression of the clinical condition (153, 196). Specifically, these theories start to fill the explanatory gap between an apparent neurochemical disturbance in the brain and the formation of complex delusions and hallucinations. The argument is that delusions arise as a result of individuals’ explanations of experienced aberrant salience. This argument is based on the role of DA in mediating both the presence or absence of reward and the salience, such as novelty, of stimuli. Increased DA neurotransmission is thought to lead to aberrant salience, that is, the direction of attention to objectively irrelevant internal or external stimuli. Delusions are by this account thought to arise through individuals’ attempts to explain this distressing experience, likely in interaction with prior experiences and beliefs, both personal and socio-cultural. While it is not clear yet which aspect of salience is particularly disturbed in schizophrenia, this work provides a promising model for understanding an important symptom of schizophrenia. Additionally, it remains unclear from this work how other symptoms of schizophrenia, such as hallucinations, thought disorder and negative symptoms arise. Because we know of numerous established similarities between high schizotypy and schizophrenia [see in Ref. (24) for a recent overview], such theories may be drawn upon for our understanding of schizotypy as well.

In sum, we conclude that there is some evidence of an association between altered DA neurotransmission and schizotypy, particularly positive schizotypy. Such work informs not only neurobiological, but also cognitive models of the schizophrenia spectrum (196) and potential neuropharmacological treatments (197).

## Future Work

The current overview illustrates the numerous and varied ways in which researchers have aimed to investigate the link between schizotypy and DA. The heterogeneity in the approaches and the sometimes conflicting results make it difficult to arrive at a clear-cut conclusion. Most of the time, we cannot directly compare results across study domains. This heterogeneity is of course frustrating but may also be constructive in providing directions as to where more work needs to be done.

Specific suggestions for future work include (1) the need to continue working on the development of an overarching theory of schizotypy (2), the assessment of the direction of effects of increasing and decreasing DA availability in high and low schizotypy of different dimensions (3), the role of concomitant illegal and legal drug use (4), the role of individuals’ genetic makeup, and (5) the identification of schizotypal markers that are truly unfavorable concerning high-risk states and those that are potentially of little psychopathological impact.

In our view, the field needs both carefully controlled laboratory experiments and studies that account for the spontaneous self-administration of psychoactive ingredients that frequently act on the DA system. For instance, there is insufficient knowledge as to why schizotypal individuals and patients with schizophrenia consume DA-sensitive drugs to a higher degree than controls (198–200). It remains to be investigated whether this drug consumption is a potential trigger for psychosis or whether such individuals medicate themselves by altering their DA systems.

Also, inconsistencies in the literature remain regarding the molecular genetics of schizotypy. Future studies are needed to provide firmer answers on the important question of the molecular genetic makeup of schizotypy. Given the advance in sample sizes in the genetics of schizophrenia and the likely small sizes of genetic effects on schizophrenia spectrum phenotypes (201), it will be important to substantially increase sample sizes in the study of molecular genetic correlates of schizotypy. A promising way to achieve appropriate sample sizes is the establishment of multi-site collaborations under the guidance of consortia.

An additional avenue for future work concerns the direct comparison between individuals with high levels of schizotypy and patients with schizophrenia. While differences between individuals with high and low schizotypy scores have been shown on numerous occasions, much less is known about differences between highly schizotypal individuals and schizophrenia patients. Such a comparison would allow the identification of factors that perhaps protect schizotypes from developing the full-blown clinical condition (24).

Finally, it should be pointed out as a limitation that this overview provides merely a qualitative discussion of the evidence of an association between DA and schizotypy. An important future contribution to this literature would be a formal meta-analysis of the size and significance of this association across different studies and methods.

## Conflict of Interest Statement

The authors declare that the research was conducted in the absence of any commercial or financial relationships that could be construed as a potential conflict of interest.
